# Supplementary material for: Phylotranscriptomics Reveals Discordance in the Phylogeny of Hawaiian Drosophila and Scaptomyza (Diptera: Drosophilidae)
Source: Mol Biol Evol. 2022 Jan 20;39(3):msac012. doi: 10.1093/molbev/msac012 (PMC8892949; doi:10.1093/molbev/msac012)
Supplement: msac012_Supplementary_Data [file msac012_supplementary_data.pdf]

Supplementary methods - Phylotranscriptomics reveals discordance in the phylogeny of Hawaiian *Drosophila* and *Scaptomyza* (Diptera: Drosophilidae)

Samuel H. Church[1]*, Cassandra G. Extavour[1,2,3]

Contents

Previous phylogenetic hypotheses 2
Phylotranscriptomic results 3
Concordance factor analysis 9
Expanded phylogenetic analysis 14
Oviposition ecology 16
Trait diversification 17
Voucher specimen photos 22
Supplementary Tables 23
References 26

[1] Department of Organismic and Evolutionary Biology, Harvard University, Cambridge, MA 02138, USA

[2] Department of Molecular and Cellular Biology, Harvard University, Cambridge, MA 02138, USA

[3] Howard Hughes Medical Institute, Chevy Chase, MD 20815

* Corresponding author

Previous phylogenetic hypotheses

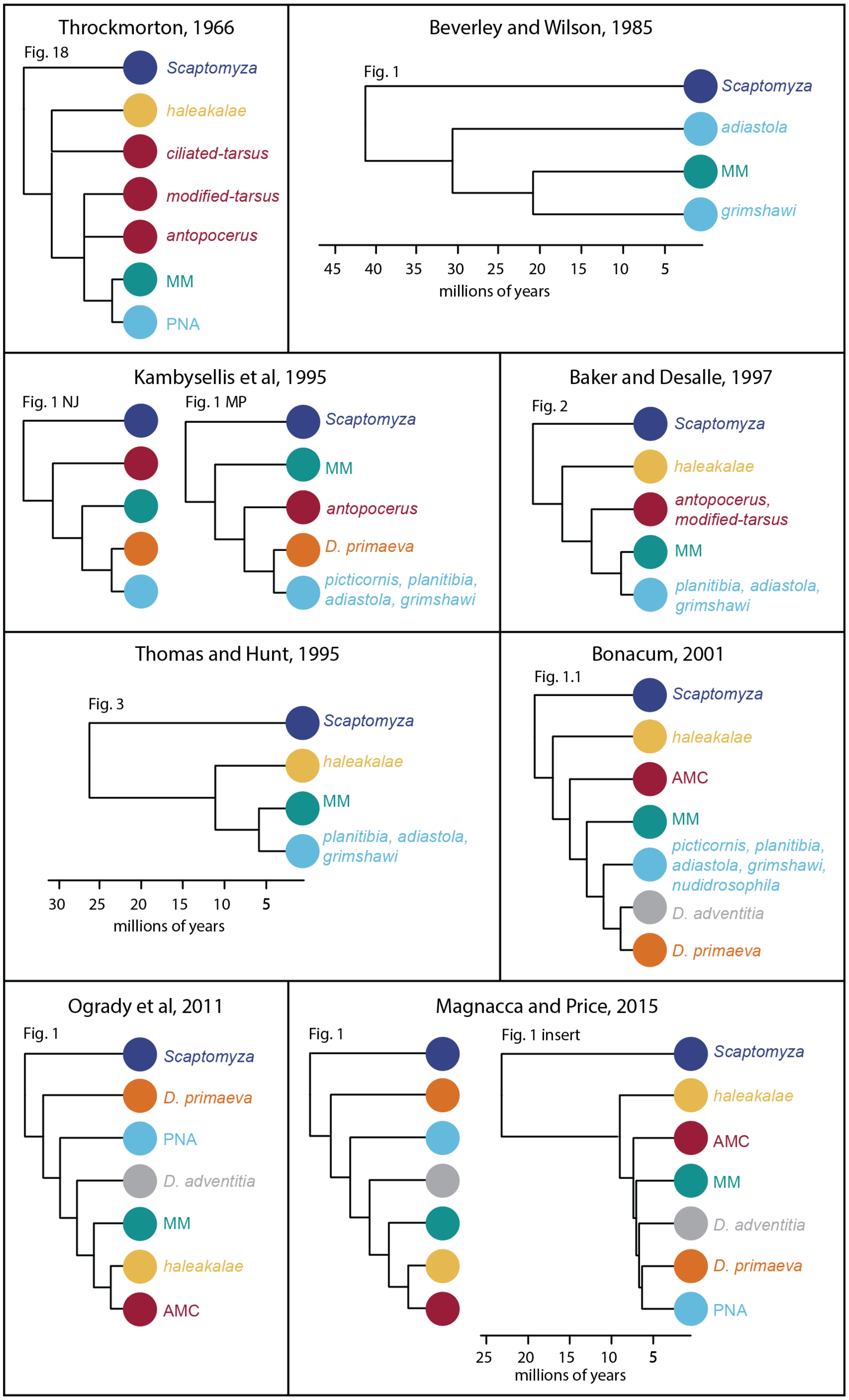

Figure S1: Selected previously published phylogenetic hypotheses for the relationships between clades of Hawaiian Drosophilidae. Figure labels indicate the figure number as originally published[1–8].

Phylotranscriptomic results

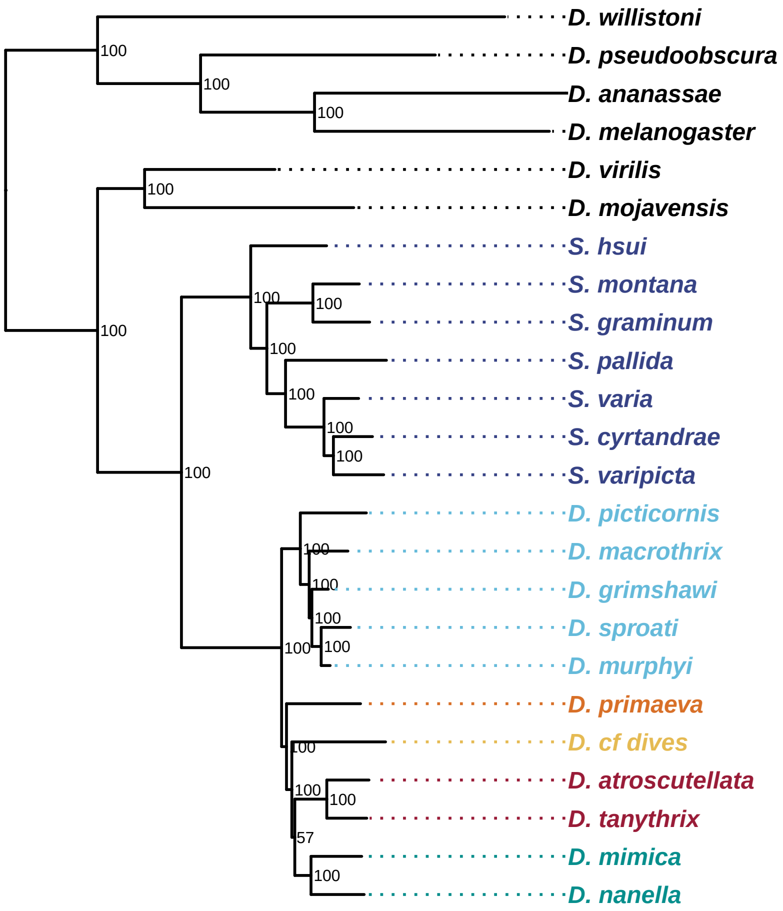

Figure S2: Most likely tree estimated using RAxML. Node labels show bootstrap values. Colors correspond to clades described in Fig. 1 and S1.

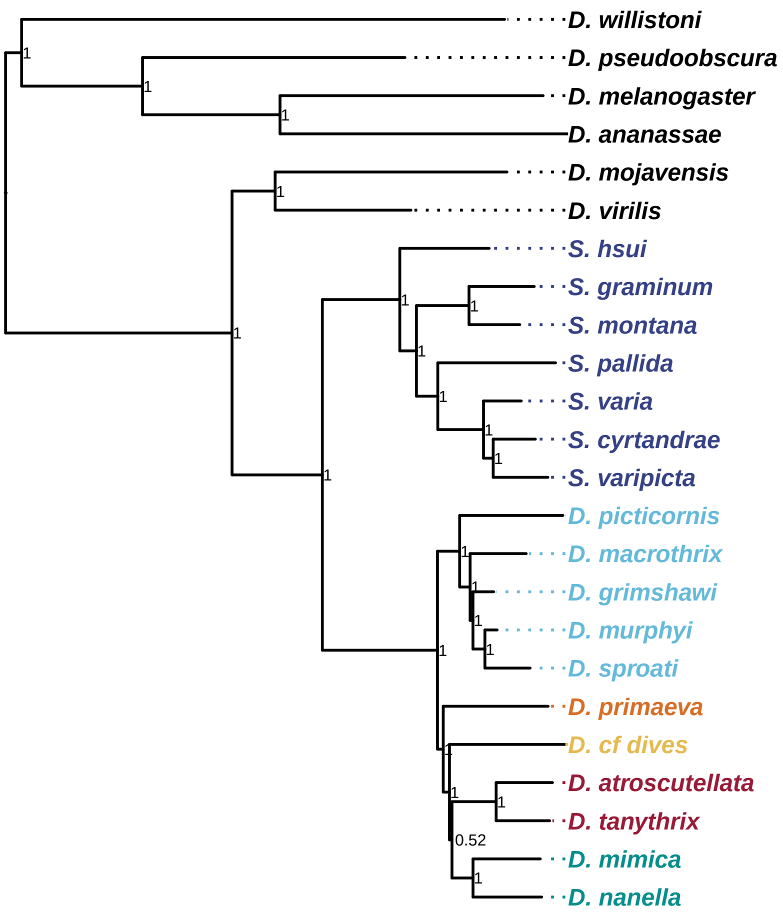

Figure S3: Consensus tree estimated using PhyloBayes. Node labels show posterior support. Colors correspond to clades described in Fig. 1 and S1.

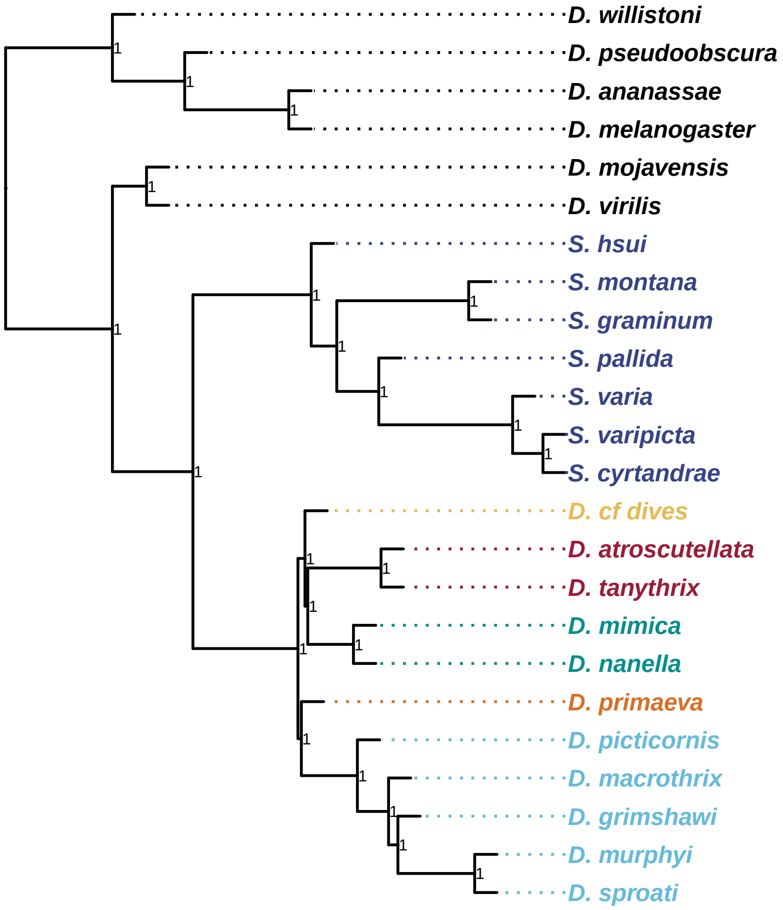

Figure S4: Coalescent tree estimated using ASTRAL. Node labels show local posterior probabilities. ASTRAL estimates branch lengths for internal nodes only, therefore tip branch lengths have been artificially set to a length of 0.5 coalescent units. Colors correspond to clades described in Fig. 1 and S1.

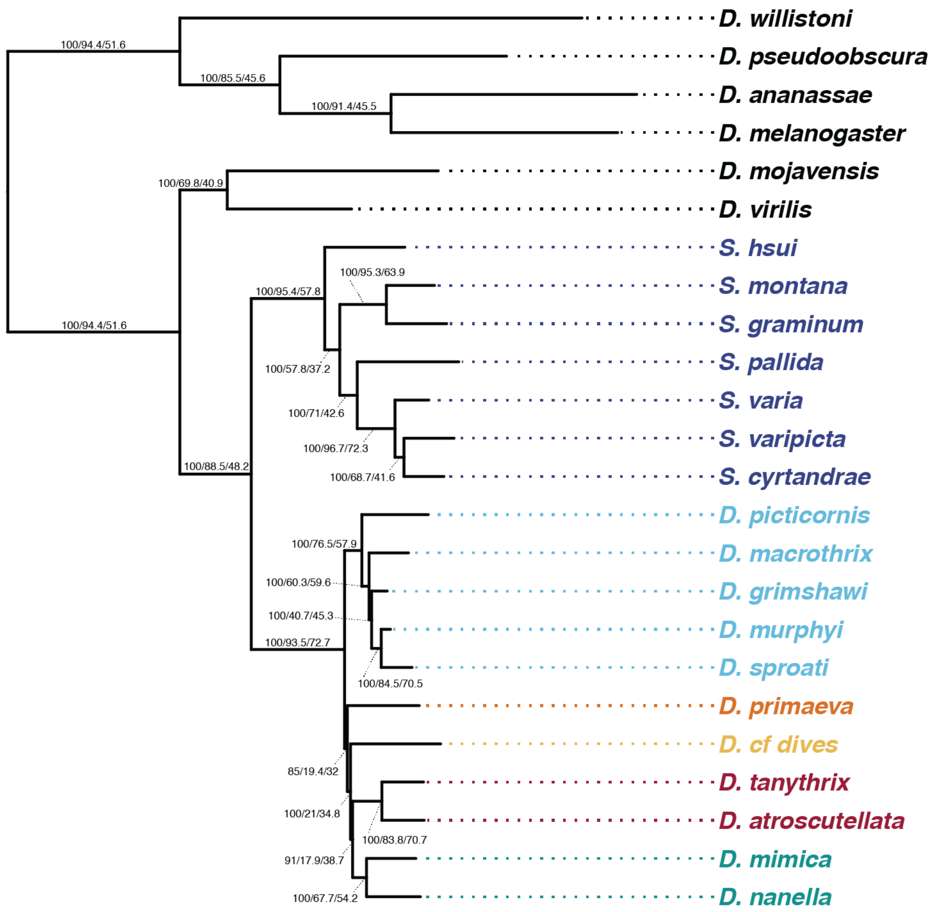

Figure S5: Most likely topology estimated using IQtree on a trimmed dataset, using a an occupancy threshold of 80%. Node labels show boostrap values / gene concordance factors / site concordance factors. Colors correspond to clades described in Fig. 1 and S1.

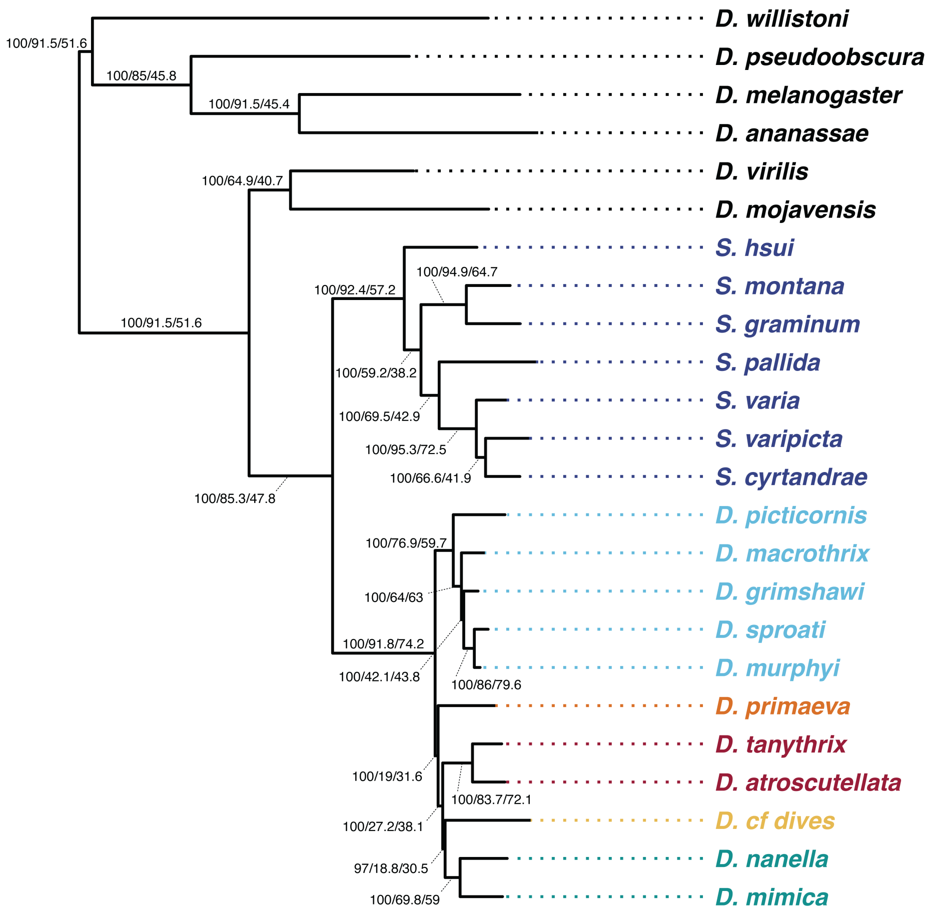

Figure S6: Most likely topology estimated using IQtree on a dataset filtered to exclude poorly aligned sequence fragments, above a 95% cutoff. Node labels show boostrap values / gene concordance factors / site concordance factors. Colors correspond to clades described in Fig. 1 and S1.

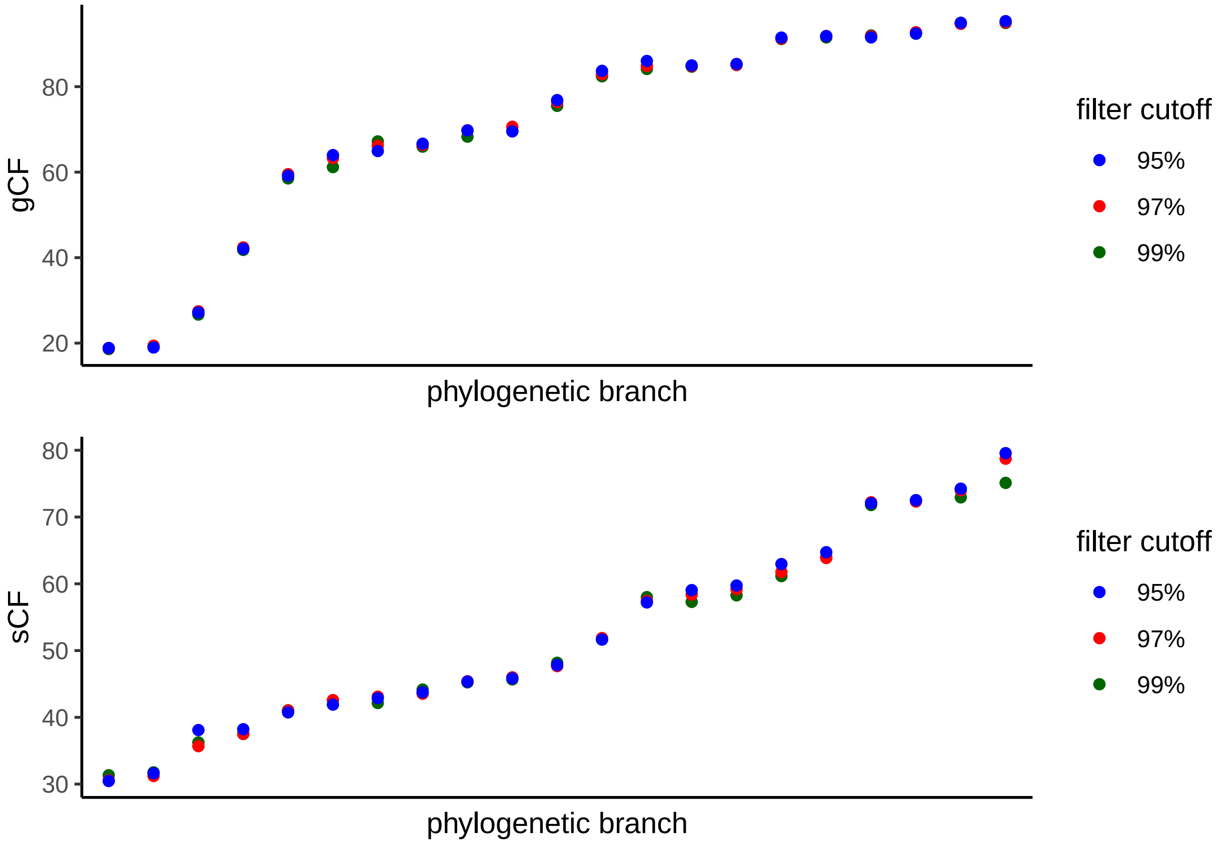

Figure S7: Comparison of concordance factors across branches using variable cutoff values to filter potentially poorly aligned sequence fragments. In both panels, the x-axis shows the 21 branches of the phylogeny, arranged from lowest concordance factor value to highest.

Concordance factor analysis

There are 210 unique internal branches across all possible topologies of Hawaiian *Drosophila* clades, when the root of the phylogeny is considered to be fixed at the base of the split between *Scaptomyza* and Hawaiian *Drosophila*, and each of the major clades is considered to be monophyletic. Each of these branches defines a relationship between four groups , and in rooted trees like those considered here, one of those groups includes the outgroup. These 210 branches can be divided into four categories:

[1] 15 branches that define the split between *Scaptomyza* and Hawaiian *Drosophila*, which differ based on the arrangement of clades on the *Drosophila* side of the branch. These have universally high gene tree concordance (minimmum of 89.38), and the small amount of variation between them can be attributed to variation in the number of informative sites.

[2] 70 branches that define a relationship that unites any two clades on one side of a branch (panel A in Figs. S8 and S9). These branches indicate support for two clades as sister to one another, and variation across these branches shows that more genes support the unification of *D. primaeva*+PNA and any two of the clades AMC, MM, and *haleakalae*, relative to other groupings.

[3] 90 branches that define a split between two clades of Hawaiian *Drosophila* and the other three (Figs. S8 and S9, panel B). Variation in support across these branches shows a marked increase in the number of genes that support AMC+MM+*haleakalae*, relative to other groupings.

[4] 35 branches that define the split at the base of the Hawaiian *Drosophila* as having one clade sister to the rest of Hawaiian *Drosophila* (or in other words, a branch separating one clade from the other four, Figs. S8 and S9, panel C). Variation in support across these branches shows that the fewest number of genes support either MM or AMC as sister to the rest.

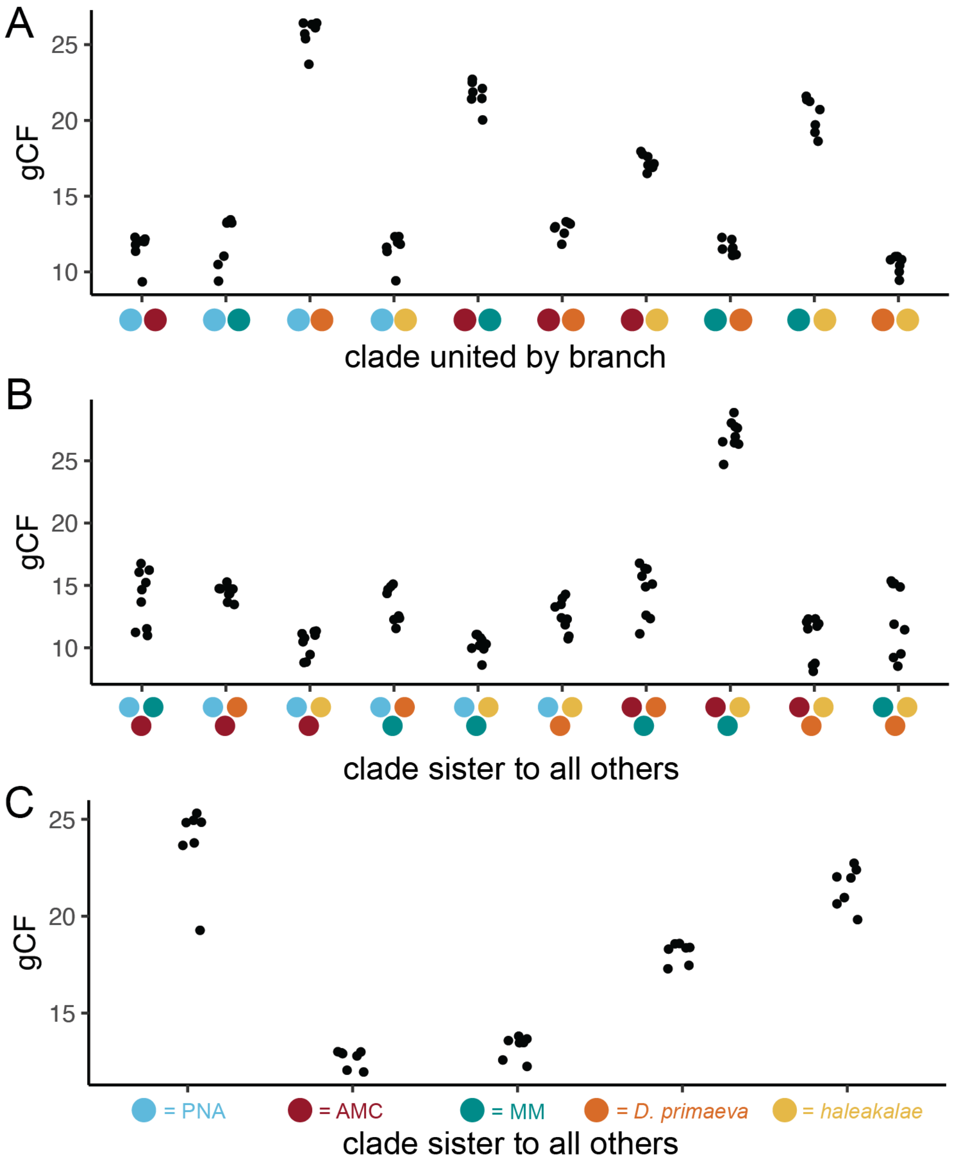

Figure S8: Gene concordance (gCF) across all possible branches.

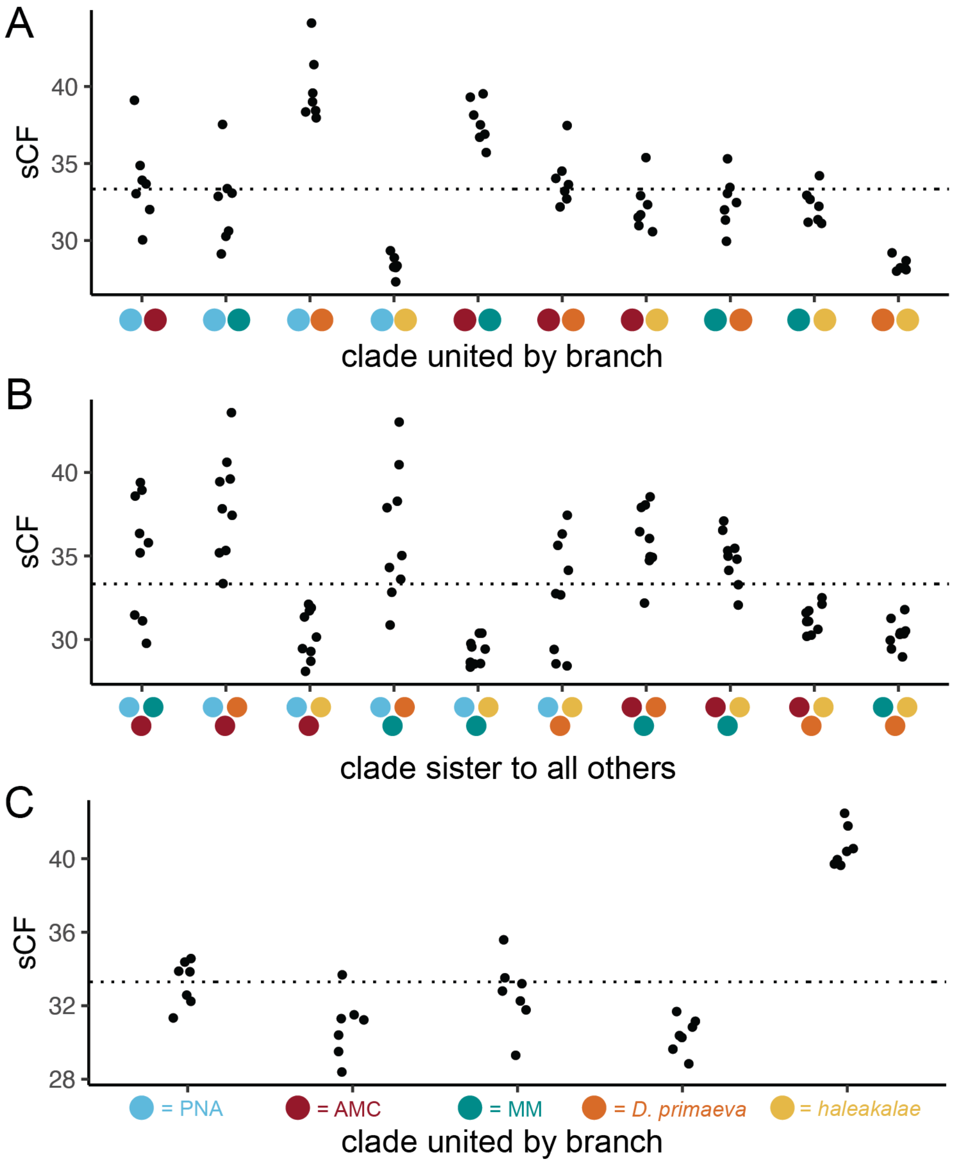

Figure S9: Site concordance (sCF) across all possible branches.

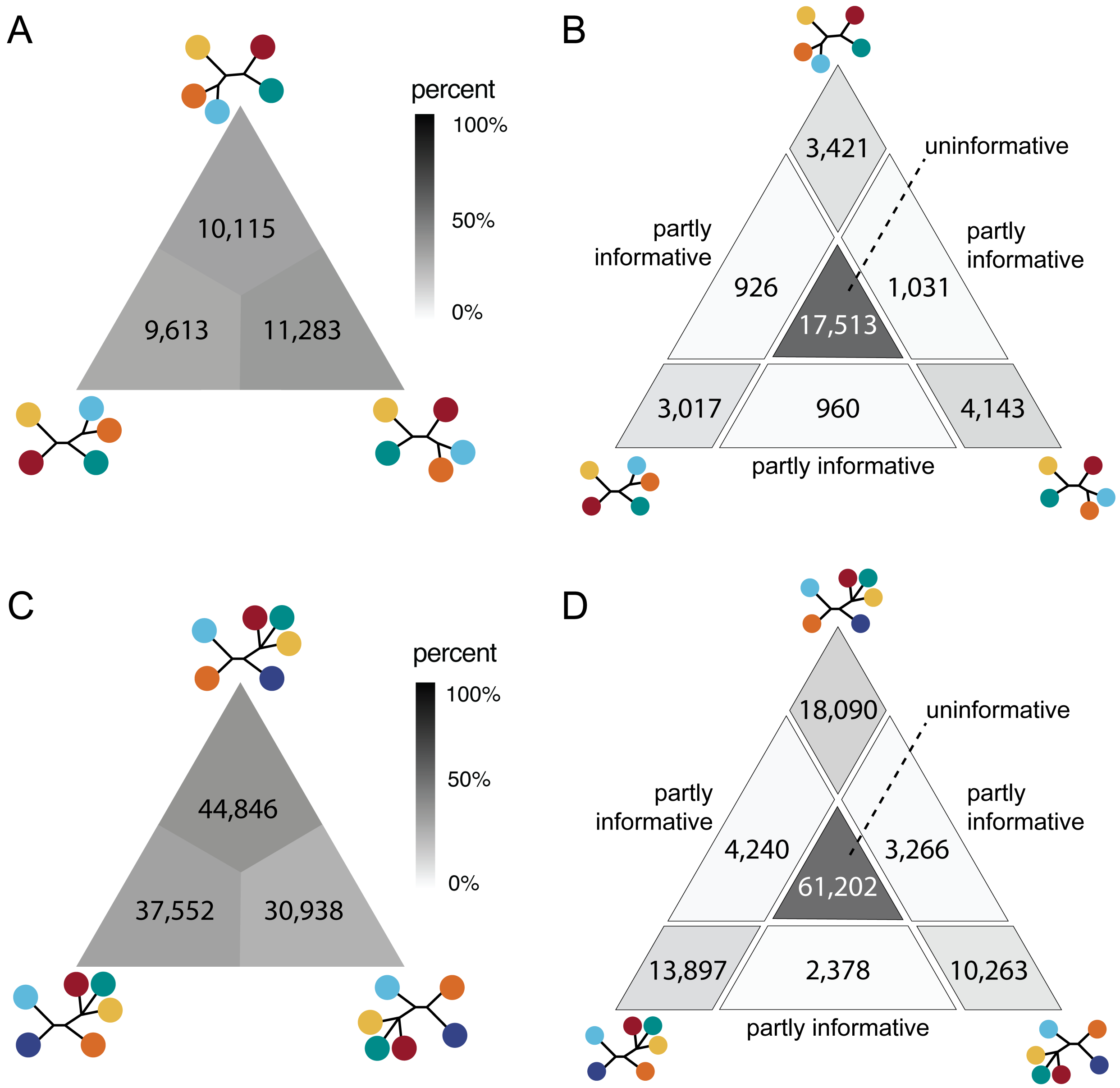

Figure S10: Results of a likelihood mapping analysis of all taxon quartets across all genes. A-B show support for the branches subtending the *haleakalae* group, relative to AMC, MM, and PNA+*D. primaeva*. C-D show support for the branches subtending the PNA group, relative to *D. primaeva*, AMC+MM+*haleakalae*, and *Scaptomyza*. Panels A and C show the number of taxon quartets that support the arrangements shown at the vertices. Panels B and D show the number of quartets that are clearly informative for these arrangements, compared to quartets that are only partly informative or uninformative in distinguishing these arrangements. Triangle panels are shaded by the percent of quartets.

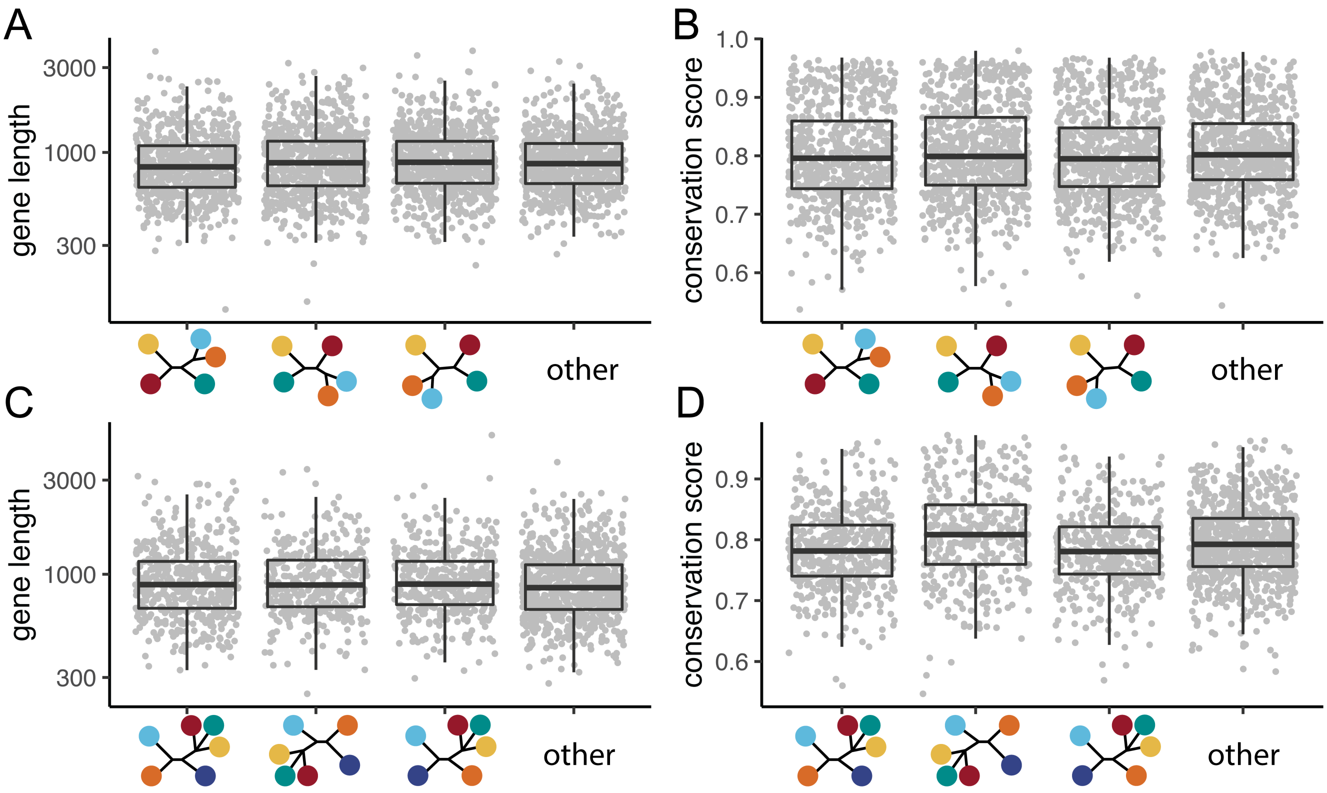

Figure S11: Distributions of genes supporting particular arrangements by gene length and evolutionary conservation. A-B show support for the branches subtending the *haleakalae* group, relative to AMC, MM, and PNA+*D. primaeva*. C-D show support for the branches subtending the PNA group, relative to *D. primaeva*, AMC+MM+*haleakalae*, and *Scaptomyza*. Boxplots show the median, quartiles, and outlier values.

Expanded phylogenetic analysis

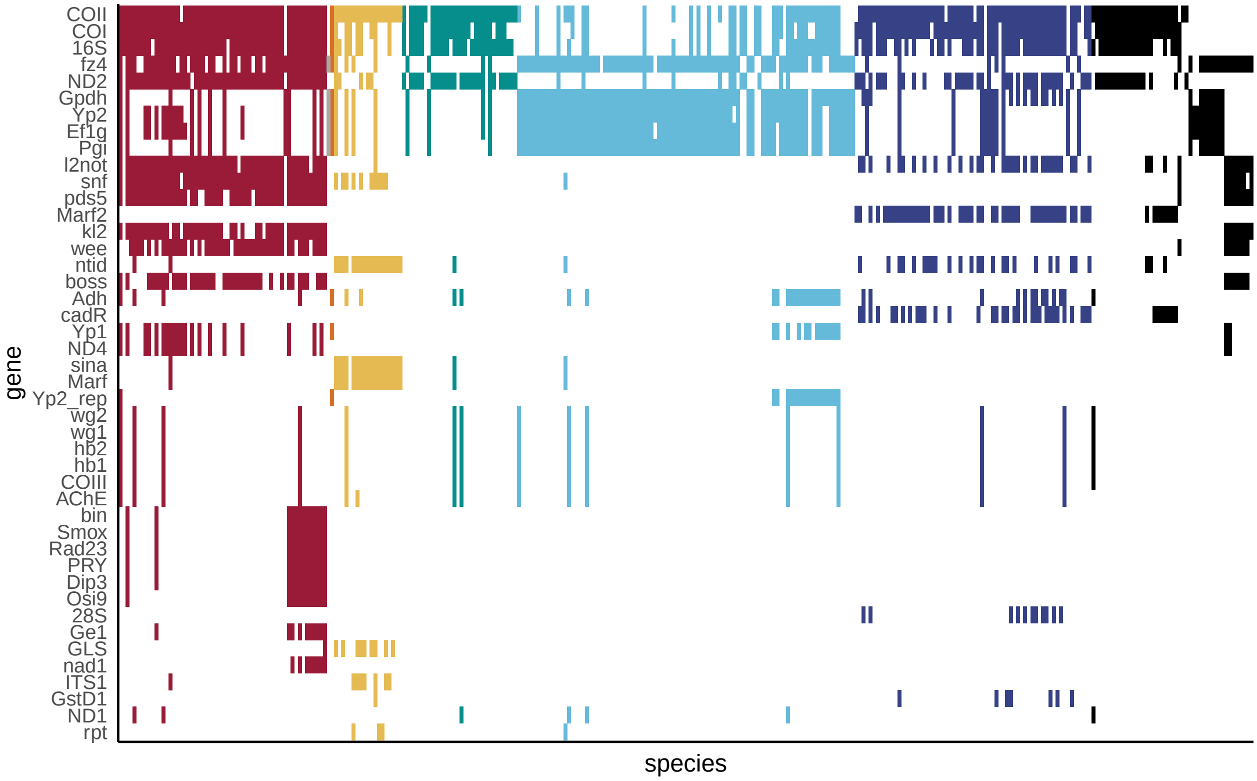

Figure S12: Occupancy matrix of genes in the expanded phylogenetic analysis using previously published mitochondrial and nuclear genetic data, ordered by high to low occupancy on the y axis and by clade, subgenus, group, and subgroup on the x axis. Colors correspond to Fig. 1 and S1, black indicates undescribed species.

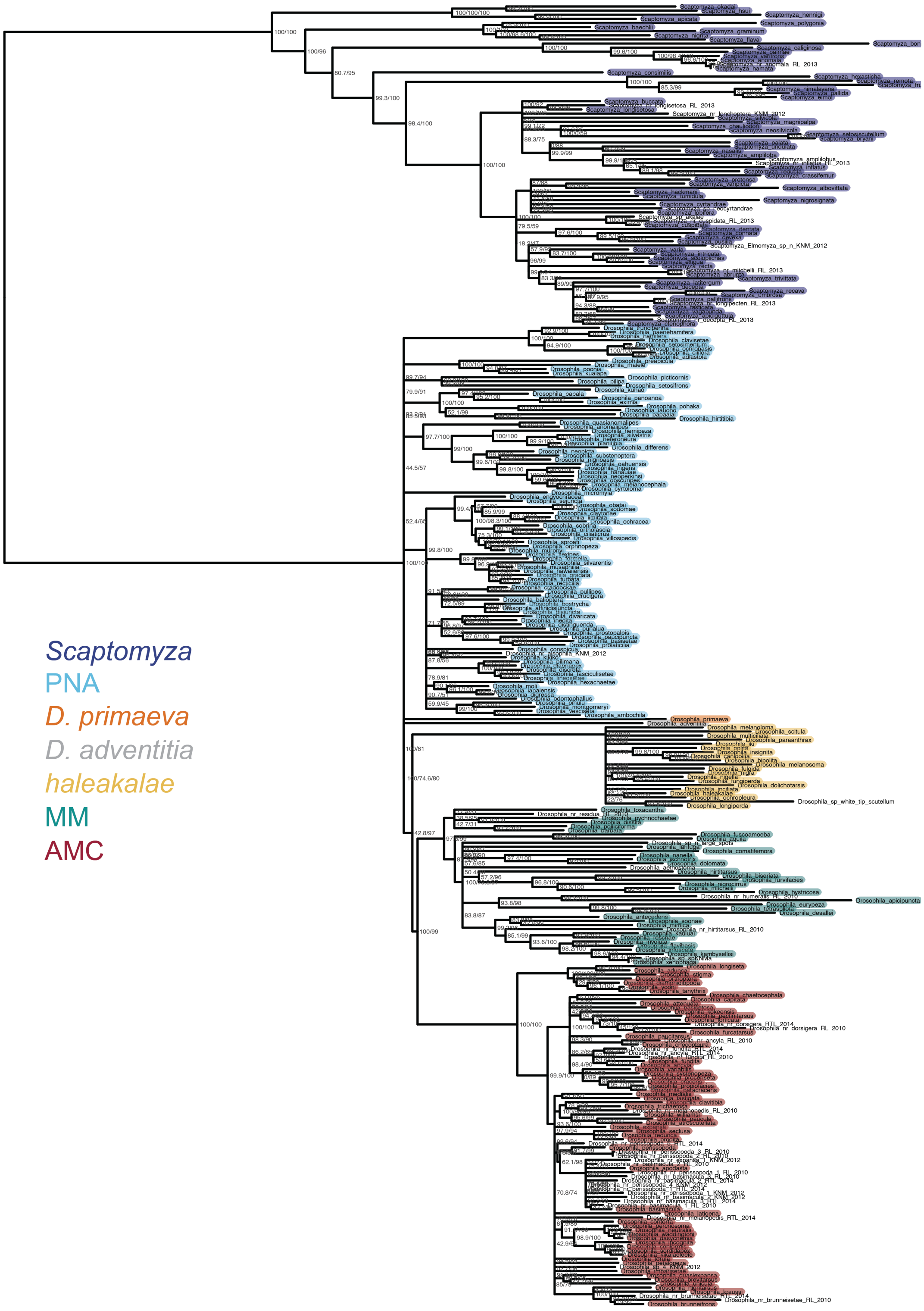

Figure S13: Most likely tree estimated with IQtree using previously published genetic data. The tree search was constrained to follow the relationships estimated using phylotranscriptomic data. Support values shown are SH-like approximate likelihood ratio test / ultrafast bootstrap. Nodes with an ultrafast bootstrap support <95 have been collapsed.

Oviposition ecology

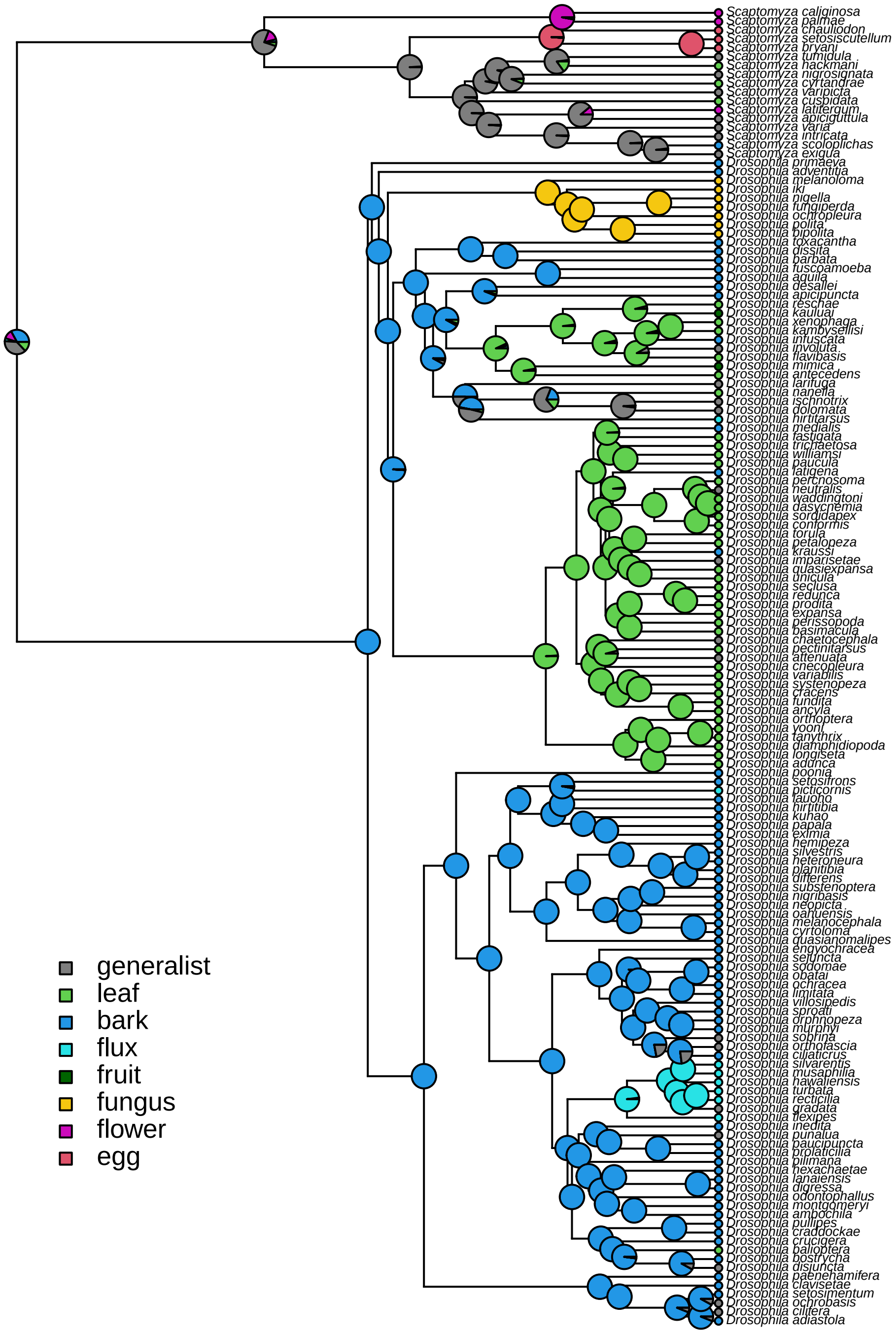

Figure S14: Ancestral state reconstruction of oviposition substrate based on rearing records[9] using stochastic character mapping. Generalist species are defined as those with any two substrates that each comprise >1/4 of rearing records, or any species without one substrate comprising more than >2/3 of rearing records[9]. 'Flux' refers to sap flux breeding, 'egg' refers to spider egg breeding.

Trait diversification

Body length, wing length, and thorax length

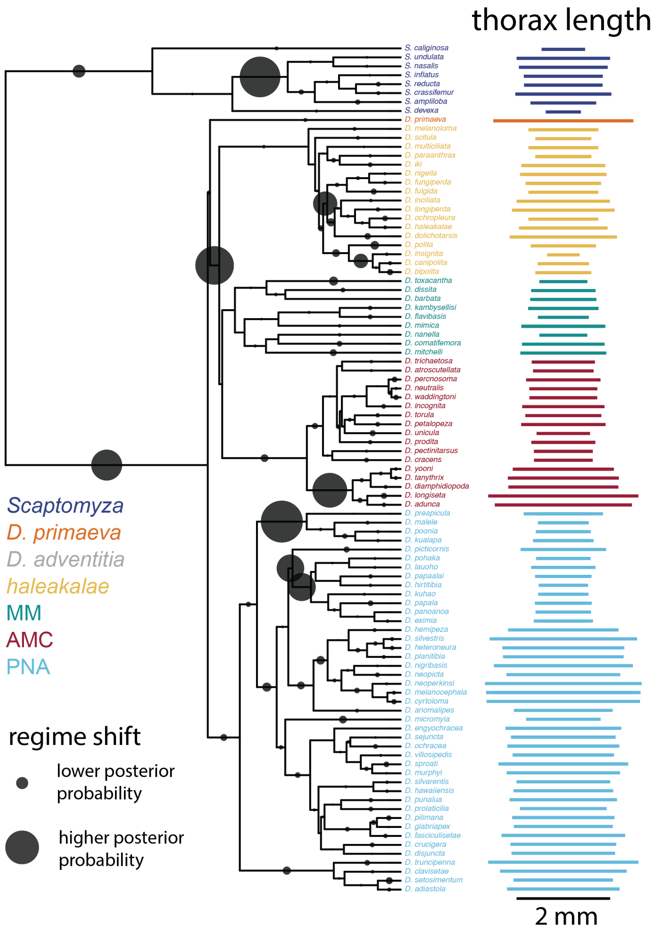

Figure S15: Model of the evolution of thorax length (mm). Data digitized from 26 publications[10–35]. Probable shifts in evolutionary regimes shown by gray circles. Larger circles indicates greater posterior probability that a shift occured on that branch. Distribution of thorax length measurements shown next to tips.

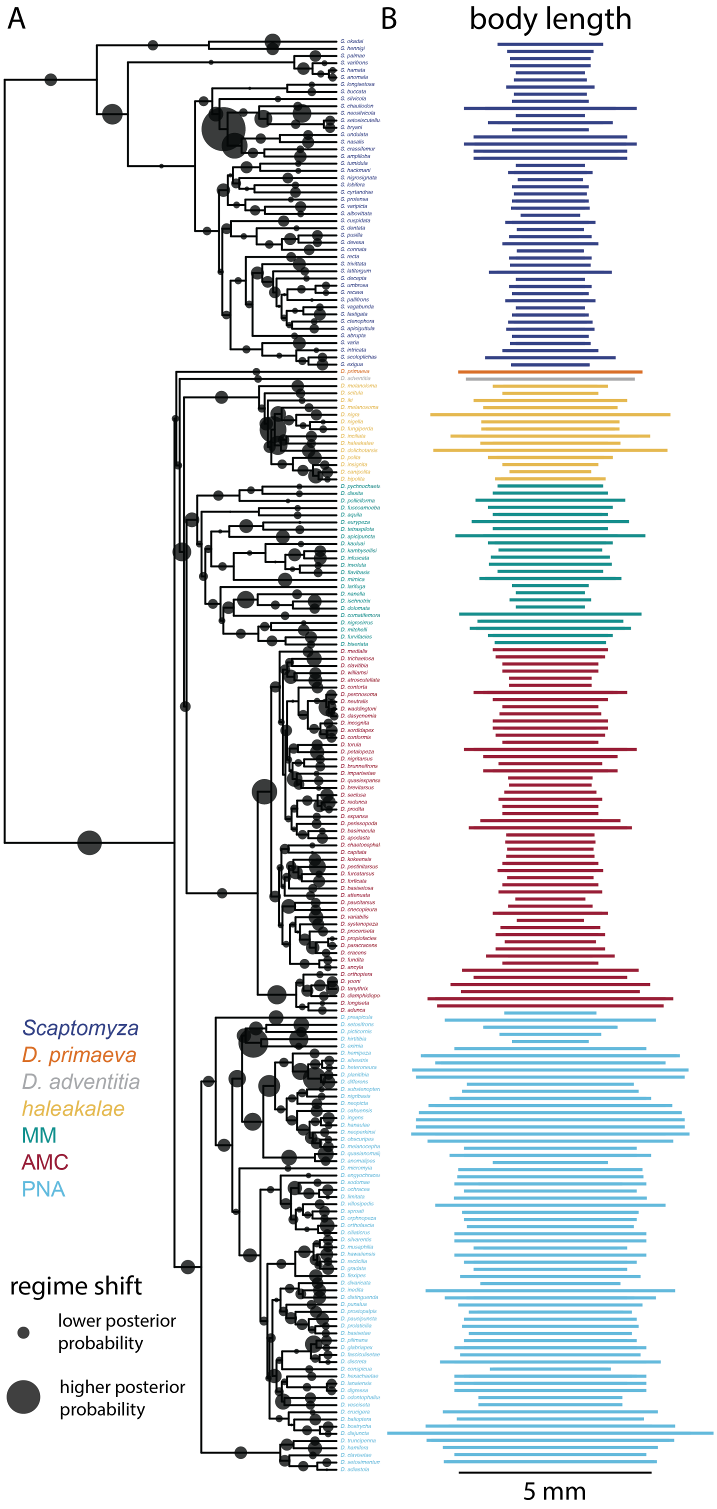

Figure S16: Model of the evolution of body length (mm). Data digitized from 26 publications[10–35]. Probable shifts in evolutionary regimes shown by gray circles. Larger circles indicates greater posterior probability that a shift occured on that branch. Distribution of body length measurements shown next to tips.

Egg size and shape

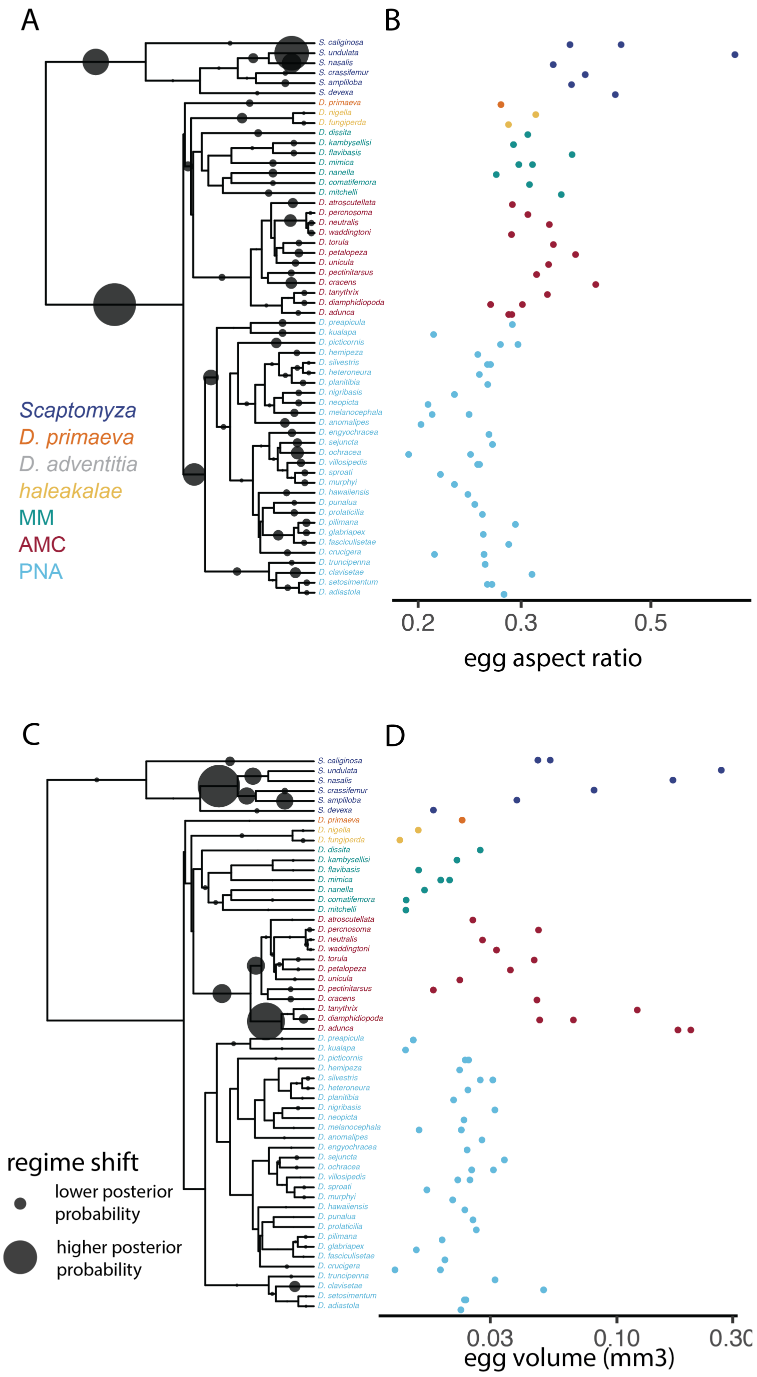

Figure S17: A and C, model of the evolution of egg volume (mm3) and aspect ratio (unitless), probable shifts in evolutionary regimes shown by gray circles. Data digitized from three publications[26,31,35]. Larger circles indicates greater posterior probability that a shift occured on that branch. B and D, Egg volume (mm3) and aspect ratio (unitless), log10 transformed.

Ovariole number

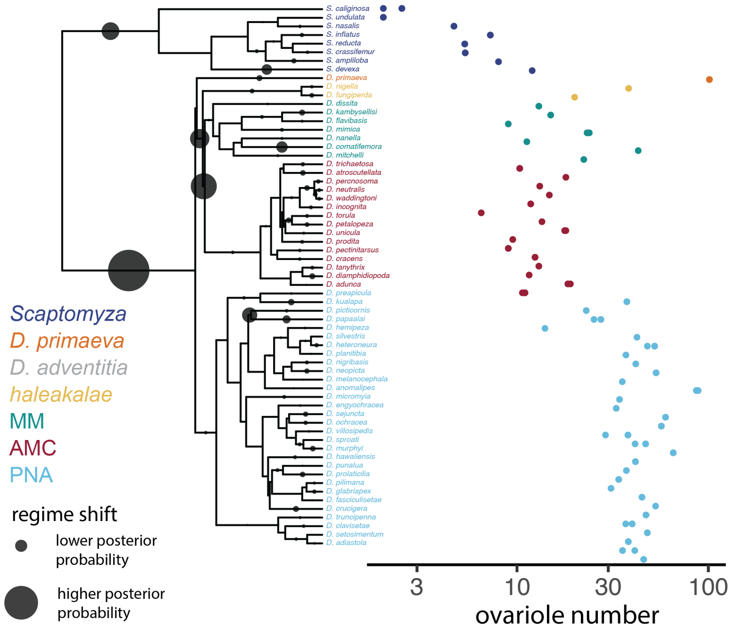

Figure S18: Model of the evolution of ovariole number, probable shifts in evolutionary regimes shown by gray circles. Data digitized from three publications[26,31,35]. Larger circles indicates greater posterior probability that a shift occured on that branch. Ovariole number, log10 transformed, shown adjacent to tips.

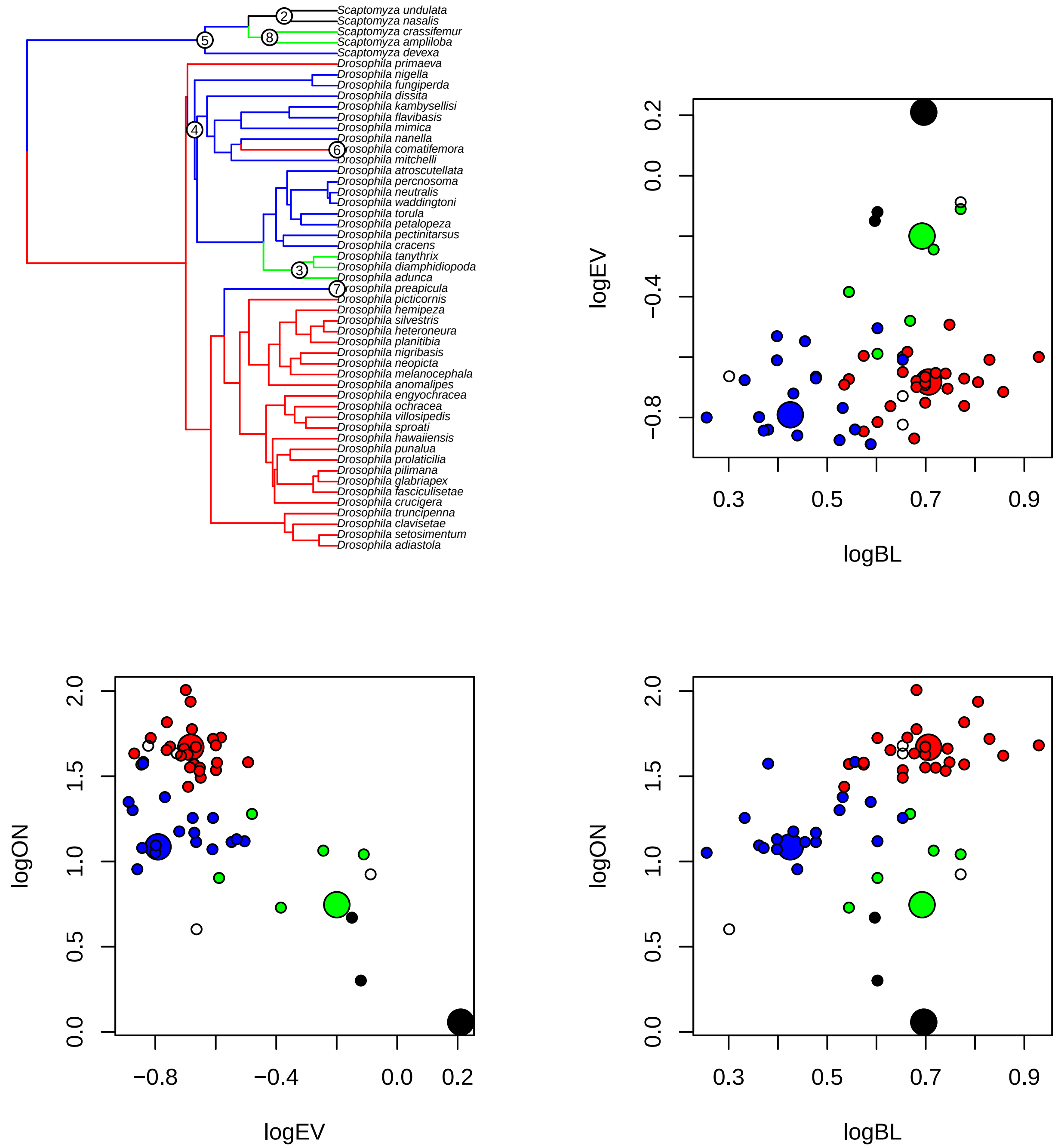

Figure S19: SURFACE estimate of convergent regime shifts in three traits. SURFACE estimates shifts in evolutionary regimes for multiple traits at once, and then assesses whether independent shifts can be combined into convergent regimes. Considering three traits (BL - body length, EV - egg volume, and ON - ovariole number, all log10 transformed, SURFACE finds evidence for eight shifts between four regimes. These can be described as a regime with high EV and low ON, seen in *Scaptomyza* species, a regime with high EV, high BL, and medium ON, seen in *Scaptomyza* and *antopocerus* species, a regime with high ON and high BL, seen in PNA species, *D. primaeva*, and *D. crassifemur*, and a regime with low BL and EV, seen in all others.

Voucher specimen photos

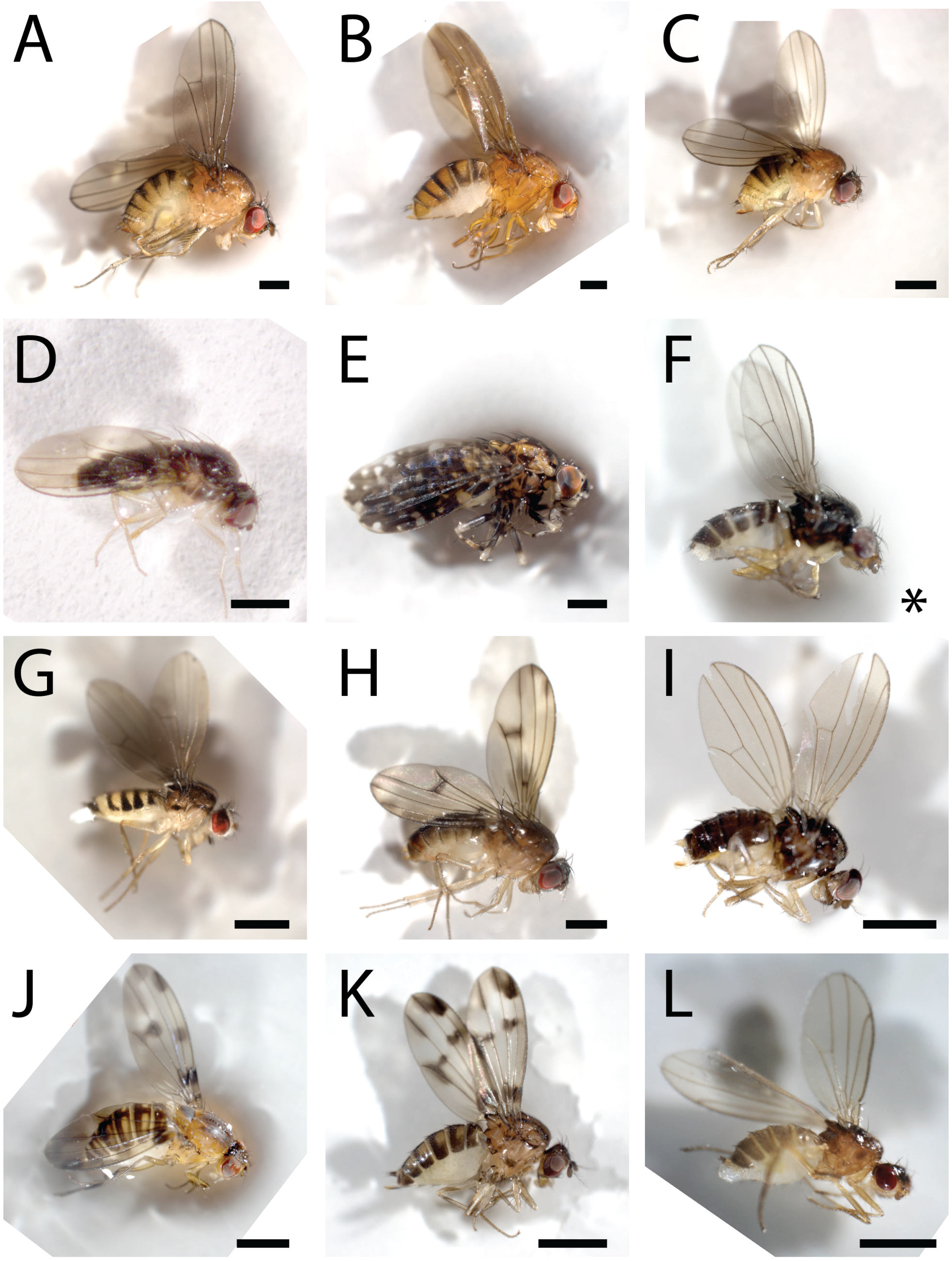

Figure S20: Photos of specimens used for transcriptome sequencing. Species are A, *D. tanythrix*, B, *D. primaeva*, C, *D. atroscutellata*, D, *D.* cf *dives*, E, *D. picticornis*, F, *S. varia*, G, *S. albovittata*, H, *D mimica*, I, *D. nanella*, J, *D. sproati*, K, *D. macrothrix*, L, *S. cyrtandrae*. Scale bar = 1 mm. Asterisk in panel F indicates scale bar failed to be recorded at the time image was captured.

Supplementary Tables

Table S1: Field collection information for transcriptome sequenced specimens.

ID	species	general site	locality	collection date	collection method	permit	GPS
16.1-1	*Drosophila cf dives*	Hawai'i Volcanoes National Park	Bird park	5/9/2016	baits	DOFAW I1012; HAVO-2017-SCI-0017	N19° 26.3512' W155° 18.2225'
040C	*Drosophila mimica*	Hawai'i Volcanoes National Park	Bird park	4/17/2017	sweeping Sapindus saponaria leaves	DOFAW I1012; HAVO-2017-SCI-0017	N19° 26.3512' W155° 18.2225'
055A	*Drosophila macrothrix*	Hawai'i Volcanoes National Park	Ola'a tract, pole 44	4/17/2017	baits	DOFAW I1012; HAVO-2017-SCI-0017	N19° 27.722' W155° 14.875'
043D	*Drosophila tanythrix*	Hawai'i Volcanoes National Park	Ola'a tract, pole 44	4/18/2017	baits	DOFAW I1012; HAVO-2017-SCI-0017	N19° 27.722' W155° 14.875'
106A	*Drosophila sproati*	Hawai'i Volcanoes National Park	Ola'a tract, pole 44	5/29/2017	baits	DOFAW I1012; HAVO-2017-SCI-0017	N19° 27.722' W155° 14.875'
025A	*Drosophila picticornis*	Koke'e State Park	Awa'awapuhi trail	4/15/2017	baits	DOFAW I1012; Koke'e state park K2017-2015; I1012; NARS special use; Kaua'i island forest reserves KPI-2017-114	N22° 08.481' W159° 38.926'
002D	*Drosophila nanella*	Koke'e State Park	Drosophila ditch	4/13/2017	sweeping Paisonia Leaves	DOFAW I1012; Koke'e state park K2017-2015; I1012; NARS special use; Kaua'i island forest reserves KPI-2017-114	N22° 04.795' W159° 40.448'
029A	*Drosophila atroscutellata*	Koke'e State Park	Nualolo trail	4/16/2017	sweeping Corynocarpus sp leaves	DOFAW I1012; Koke'e state park K2017-2015; I1012; NARS special use; Kaua'i island forest reserves KPI-2017-114	N22° 07.801' W159° 39.617'
020A	*Scaptomya varipicta*	Koke'e State Park	Nualolo trail	4/15/2017	sweeping Cheirodendron sp. leaves	DOFAW I1012; Koke'e state park K2017-2015; I1012; NARS special use; Kaua'i island forest reserves KPI-2017-114	N22° 07.801' W159° 39.617'
008D	*Drosophila primaeva*	Koke'e State Park	Pihea trail	4/14/2017	baits	DOFAW I1012; Koke'e state park K2017-2015; I1012; NARS special use; Kaua'i island forest reserves KPI-2017-114	N22° 08.799' W159° 37.074'
CFB	*Scaptomya varia*	Koke'e State Park	Pihea trail	4/14/2017	collected rotting Clermontia sp flowers	DOFAW I1012; Koke'e state park K2017-2015; I1012; NARS special use; Kaua'i island forest reserves KPI-2017-114	N22° 08.799' W159° 37.074'
088B	*Scaptomya cyrtandrae*	Stainback Highway	Army road - west	5/29/2017	on Cyrtandra platyphylla	DOFAW I1012; NARS special use; Hawai'i island forest reserve access permit	N19° 33.615' W155° 15.010'

Table S2: DNA barcoding for identification of females.

individual	sample	species match	reference male	external reference sequence	barcode sequence used for final identification	notes
029A	029Atxt	*D. atroscutellata*	yes	yes	COII	
088B	088Bb	*S. cyrtandrae*	none	yes	COII	
040C	040Ctxt	*D. mimica*	yes	yes	16S	
002D	002Dtxt	*D. nanella*	yes	yes	16S, COII	
106A	106Atxt	*D. sproati*	none	none	COII	matched to other females, morphology is distinctive for females in this species
043D	043Dtxt	*D.tanythrix*	yes	yes	COII	barcode sequences for D. cognata and D. yooni males suggest hidden complexity in this group
CFB	CFBb	*S. varia*	yes	yes	COI	
020A	020Atxt	*S. varipicta*	yes	yes	COII	
16.1-1	16.1.4	*D. cf dives*	none	none	16S, COII	found no matching reference sequence and no males were caught

Table S3: Sequencing read counts

species	individual	sample	tissue	Reads - June 2018	Reads - July 25, 2018	Reads - Nov 27, 2018	Total reads
S. varia	CFB	CFBb	carcass		6,672,052		6,672,052
S. varia	CFB	CFBn	head		6,311,203		6,311,203
S. varia	CFB	CFBo	ovary		12,672,693		12,672,693
S. cyrtandrae	088B	088Bb	carcass		9,166,453		9,166,453
S. cyrtandrae	088B	088Bn	head		8,796,864		8,796,864
S. cyrtandrae	088B	088Bo	ovary		9,763,204		9,763,204
D. sproati	106A	106Atxt	whole fly	7,432,370	6,026,569	10,237,744	23,696,683
D. atroscutellata	029A	029Atxt	whole fly	12,520,922	7,801,177	19,925,042	40,247,141
D. macrothrix	055A	055Atxt	whole fly	10,336,762	10,313,394	19,643,740	40,293,896
D. mimica	040C	040Ctxt	whole fly	9,471,290	8,887,955	15,751,511	34,110,756
D. nanella	002D	002Dtxt	whole fly	10,833,205	7,350,705	17,211,801	35,395,711
D. picticornis	025A	025Atxt	whole fly	10,085,602	10,177,523	16,172,455	36,435,580
D. primaeva	008D	008Dtxt	whole fly	9,129,075	7,583,577	13,937,132	30,649,784
D. tanythrix	043D	043Dtxt	whole fly	11,293,054	8,833,878	15,667,639	35,794,571
S. varipicta	020A	020Atxt	whole fly	7,690,349	8,004,757	14,380,332	30,075,438
D. cf dives	16.1-1	16.1.1	ovary	16,808,131			16,808,131
D. cf dives	16.1-1	16.1.2	head	18,215,227			18,215,227
D. cf dives	16.1-1	16.1.4	body		8,773,601		8,773,601

Table S4: Genome source information[36,37]

species	publication	genome source
D. virilis	FlyBase	flybase.org - file dvir-all-transcript-r1.07.fasta
D. grimshawi	FlyBase	flybase.org - file dgri-all-transcript-r1.05.fasta
D. melanogaster	FlyBase	flybase.org - file dmel-all-transcript-r6.29.fasta
D. willistoni	FlyBase	flybase.org - file dwil-all-transcript-r1.05.fasta
D. mojavensis	FlyBase	flybase.org - file dmoj-all-transcript-r1.04.fasta
D. pseudoobscura	FlyBase	flybase.org - file dpse-all-transcript-r3.04.fasta
D. annanassae	FlyBase	flybase.org - file dana-all-transcript-r1.06.fasta
D. murphyi	Kim et al, 2020	https://web.stanford.edu/~bkim331/files/genomes/ - accessed January 2021
S. pallida	Kim et al, 2020	https://web.stanford.edu/~bkim331/files/genomes/ - accessed January 2021
S. hsui	Kim et al, 2020	https://web.stanford.edu/~bkim331/files/genomes/ - accessed January 2021
S. graminum	Kim et al, 2020	https://web.stanford.edu/~bkim331/files/genomes/ - accessed January 2021
S. montana	Kim et al, 2020	https://web.stanford.edu/~bkim331/files/genomes/ - accessed January 2021

Table S5: Oviposition substrate categories described in rearing records[9]

substrate category	original substrate listed
leaf	leaf
leaf	leaf axil
leaf	leaf base
leaf	live leaf
leaf	petiole
leaf	frond
bark	rachis
bark	bark
bark	stem
bark	wood
bark	root
bark	branch
bark	shoot
misc	frass
fungus	fungus
fruit	fruit
fruit	pod
egg	spider egg
flux	flux
flux	soil
flower	flower

Table S6: Effective size of bayou analyses on trait regimes.

trait	variable	effective size
body length	k	791.4
body length	lnL	401.9
egg aspect ratio	k	1302.1
egg aspect ratio	lnL	78.8
egg volume	k	1378.5
egg volume	lnL	89.6
ovariole number	k	1312.5
ovariole number	lnL	277.0
thorax length	k	1399.5
thorax length	lnL	37.6
wing length	k	378.4
wing length	lnL	221.9

References

1. Throckmorton, L. H. The relationships of the endemic Hawaiian Drosophilidae. *University of Texas Publications* **6615**, 335–396 (1966).
2. Beverley, S. M. & Wilson, A. C. Ancient origin for Hawaiian Drosophilinae inferred from protein comparisons. *Proceedings of the National Academy of Sciences* **82**, 4753–4757 (1985).
3. Kambysellis, M. P. *et al.* Pattern of ecological shifts in the diversification of Hawaiian *Drosophila* inferred from a molecular phylogeny. *Current Biology* **5**, 1129–1139 (1995).
4. Thomas, R. H. & Hunt, J. A. The molecular evolution of the alcohol dehydrogenase locus and the phylogeny of Hawaiian *Drosophila*. *Molecular Biology and Evolution* **8**, 687–702 (1991).
5. Baker, R. H. & DeSalle, R. Multiple sources of character information and the phylogeny of Hawaiian drosophilids. *Systematic Biology* **46**, 654–673 (1997).
6. Bonacum, J. *PhD Thesis:* Molecular systematics of the Hawaiian Drosophilidae. (Yale University, 2001).
7. O'Grady, P. M. *et al.* Phylogenetic and ecological relationships of the Hawaiian *Drosophila* inferred by mitochondrial DNA analysis. *Molecular Phylogenetics and Evolution* **58**, 244–256 (2011).
8. Magnacca, K. N. & Price, D. K. Rapid adaptive radiation and host plant conservation in the Hawaiian picture wing *Drosophila* (Diptera: Drosophilidae). *Molecular Phylogenetics and Evolution* **92**, 226–242 (2015).
9. Magnacca, K. N., Foote, D. & O'Grady, P. M. A review of the endemic Hawaiian Drosophilidae and their host plants. *Zootaxa* **1728**, 1–58 (2008).
10. Grimshaw, P. & Speiser, P. Part. IL. Diptera. *Fauna Hawaiiensis* **3**, 79–86 (1901).
11. Grimshaw, P. Diptera. *Fauna Hawaiiensis* **3**, 86 (1902).
12. Knab, F. Drosophilidae with parasitic larvae. *Insecutor Inscitiae Menstruus* **2**, 165–169 (1914).
13. Bryan, E. A review of the Hawaiian Diptera, with descriptions of new species. *Proceedings of the Hawaiian Entomological Society* **VIII**, 399–457 (1934).
14. Bryan JR, E. H. Key to the Hawaiian Drosophilidae and descriptions of new species. *Proceedings of the Hawaiian Entomological Society* **10**, 25–42 (1938).
15. Wirth, W. Two new spider egg predators from the Hawaiian islands (Diptera: Drosophilidae). *Proceedings of the Hawaiian Entomological Society* **14**, 415–417 (1952).
16. Hackman, W. On the genus *Scaptomyza* Hardy (Dipt., Drosophilidae) with descriptions of new species from various parts of the world. *Acta Zoologica Fennica* **97**, 1–73 (1959).
17. Hardy, D. *Diptera: Cyclorrhapha II, Series Schizophora Section Acalypterae I. Family Drosophilidae.* vol. 12 (University of Hawai'i Press, 1965).
18. Hardy, D. E. Descriptions and notes on Hawaiian Drosophilidae (Diptera). *Studies in Genetics* 195–244 (1966).
19. Hardy, D. E. & Kaneshiro, K. Y. New picture-winged *Drosophila* from Hawaii. *Studies in Genetics* **4**, 171–261 (1968).
20. Hardy, D. E. & Kaneshiro, K. Y. Descriptions of new Hawaiian *Drosophila*. *University of Texas Publications* **6918**, 39–54 (1969).
21. Hardy, D. Notes on Hawaiian "idiomyia" (*Drosophila*). *Studies in Genetics V* **6918**, (1969).
22. Hardy, D. E. & Kaneshiro, K. Y. New picture-winged *Drosophila* from Hawaii, part II.(Drosophilidae, Diptera). *Studies in Genetics VI* **7103**, (1971).
23. Hardy, D. & Kaneshiro, K. New picture-winged *Drosophila* from Hawaii, part III (Drosophilidae, Diptera). *Studies in Genetics VII* **7213**, (1972).

24. Hardy, D. Review of the Hawaiian *Drosophila* (antopocerus) Hardy [insects]. *Proceedings Entomological Society of Washington* **79**, (1977).

25. Hardy, D. & Kaneshiro, K. Y. A review of the modified tarsus species group of Hawaiian *Drosophila* (Drosophilidae: Diptera) i. The "split-tarsus" subgroup. **23**, 71–90 (1979).

26. Kambysellis, M. & Heed, W. Studies of oogenesis in natural populations of Drosophilidae. I. Relation of ovarian development and ecological habitats of the Hawaiian species. *The American Naturalist* **105**, 31–49 (1971).

27. Hardy, D. & Kaneshiro, K. Y. Studies in Hawaiian *Drosophila*, miscellaneous new species, no. i. *Proceedings of the Hawaiian Entomological Society* **22**, (1975).

28. O'Grady, P., Val, F. do, Hardy, D. E. & Kaneshiro, K. The rustica species group of Hawaiian *Drosophila* (Diptera: Drosophilidae). *Pan Pacific Entomologist* **77**, 254–260 (2001).

29. Hardy, D. E., Kaneshiro, K., Val, F. & O'Grady, P. Review of the haleakalae species group of Hawaiian *Drosophila* (Diptera: Drosophilidae). *Bishop Museum Bulletin in Entomology* **9**, 1–88 (2001).

30. O'Grady, P., Kam, M., Val, F. do & Perreira, W. Revision of the *Drosophila* mimica subgroup, with descriptions of ten new species. *Annals of the Entomological Society of America* **96**, 12–38 (2003).

31. Starmer, W. T. *et al.* Phylogenetic, geographical, and temporal analysis of female reproductive trade-offs in Drosophilidae. *Evolutionary Biology* 139–171 (2003).

32. Magnacca, K. N. & O'Grady, P. M. Revision of the 'nudidrosophila'and 'ateledrosophila' species groups of Hawaiian *Drosophila* (Diptera: Drosophilidae), with descriptions of twenty-two new species. *Systematic Entomology* **33**, 395–428 (2008).

33. Magnacca, K. N. & Grady, P. M. Revision of the modified mouthparts species group of Hawaiian *Drosophila* (Diptera: Drosophilidae): The ceratostoma, freycinetiae, semifuscata, and setiger subgroups, and unplaced species. (2009).

34. Craddock, E. M., Gall, J. G. & Jonas, M. Hawaiian *Drosophila* genomes: Size variation and evolutionary expansions. *Genetica* **144**, 107–124 (2016).

35. Sarikaya, D. P. *et al.* Reproductive capacity evolves in response to ecology through common changes in cell number in Hawaiian *Drosophila*. *Current Biology* **29**, 1877–1884 (2019).

36. Larkin, A. *et al.* FlyBase: Updates to the *Drosophila* melanogaster knowledge base. *Nucleic Acids Research* **49**, D899–D907 (2021).

37. Kim, B. Y. *et al.* Highly contiguous assemblies of 101 drosophilid genomes. *Elife* **10**, e66405 (2021).
